# Diffuse Pulmonary Ossification (DPO) in the Setting of Firefighter Occupation

**DOI:** 10.7759/cureus.75673

**Published:** 2024-12-13

**Authors:** Roya Tawkaliyar, Arun Adlakha

**Affiliations:** 1 Clinical Research, Edward Via College of Osteopathic Medicine, Spartanburg, USA; 2 Pulmonology, Piedmont Medical Center, Rock Hill, USA

**Keywords:** dendriform pulmonary ossification, diffuse pulmonary ossification, fibrosis, linear reticulations, lung calcifications

## Abstract

A 76-year-old man with a past occupational history as a firefighter and construction worker presented at an urgent care center with signs and symptoms of chronic dry cough, exertional dyspnea, and fatigue. His initial chest X-ray showed interstitial thickening in the middle and lower lobes with pulmonary infiltrates bilaterally. The patient was treated with an outpatient course of antibiotics. A subsequent high-resolution computed tomography (HRCT) scan of the patient’s lungs revealed extensive peripheral, bibasilar linear reticulations, interstitial thickening, and subpleural cystic changes, consistent with advanced pulmonary fibrosis. Also noted were diffuse calcifications within the areas of fibrosis. Based on the HRCT findings and negative laboratory tests, the patient was diagnosed with diffuse pulmonary ossification (DPO), the dendriform type. DPO is a rare heterotrophic lung disease characterized by the formation of bone in the lung parenchyma and alveolar spaces. It is categorized as nodular and dendriform; dendriform is associated with chronic respiratory diseases, with characteristics of ramifying spicules of mature bone laid in a serpentine fashion along the alveolar septa, containing bone marrow elements. This case shows a rare form of pulmonary ossification in the setting of chronic fibrotic interstitial lung disease. Interestingly, our patient had a past occupational history as a firefighter with chronic inhalation exposure to particulate matters such as chemicals, smoke, fumes, combustible trash, silica, and alumina, which may all have likely played a role in the pathogenesis of DPO.

## Introduction

Diffuse pulmonary ossification (DPO) is a rare lung disease characterized by ectopic bone formation within the lung parenchyma. DPO was initially defined by German anatomist and pathologist Hubert von Luschka in 1856, during a postmortem exam that showed evidence of widespread metaplastic bone in the lungs [1]. DPO can arise idiopathically or secondary to cardiovascular, chronic respiratory, or other diseases [2]. The ossification present in DPO can be subdivided into two forms: dendriform pulmonary ossification and nodular pulmonary ossification [1-5]. Dendriform pulmonary ossification has characteristics of ramifying spicules of mature bone laid in a serpentine fashion primarily along the alveolar septa, with association to fat or bone marrow elements, identified in certain cases [1]. In addition, the dendriform is primarily seen in patients with diffuse interstitial lung disease [2] or with a background of chronic lung diseases [1]. Nodular pulmonary ossification has characteristics of a round and well-circumscribed outline with the involvement of alveolar spaces [1]. The nodular form is seen in cases of passive pulmonary congestion, especially in mitral stenosis [1,5]. We conducted a literature review and observed no reported cases of DPO associated with a background occupation as a firefighter. Herein, we present a case of DPO in a 76-year-old man with a previous occupational history as a firefighter, with exposure to a multitude of inhaled respiratory insults and pollutants.

## Case presentation

A 76-year-old White male patient presented to a local urgent care center with signs and symptoms of acute bronchitis. His chest X-ray revealed bilateral, peripheral, and reticular pulmonary infiltrates with interstitial thickening, predominantly in the middle and lower lung zones. He was treated with a course of antibiotics and referred to our pulmonology clinic for further evaluation and treatment.

The patient had an ongoing dry cough, exertional dyspnea, and fatigue for the last several years. He had a past medical history of arthritis, acid reflux, hypothyroidism, diabetes mellitus (DM), hypertension, hyperlipidemia, anxiety, and allergic rhinitis. He had no known history of collagen vascular disease, sarcoidosis, asbestos exposure, hypersensitivity pneumonitis, or tuberculosis contact. The patient denied any presence of birds or chickens at home. He was a former smoker with a 20-pack-year smoking history; he quit smoking 40 years ago. He was not on oxygen therapy.

He worked in a fire department for over 20 years. On several occasions, he had to extinguish fires at the city’s residential and commercial trash dump. He was exposed to commercial/residential combustible trash mixtures. On other occasions, he had to put out fires in the industrial buildings, secondary to chemical leaks. He was exposed to different inflammable chemical fumes. At one point, he was called out to extinguish a fire containing gasoline and petroleum products. Another time, while fighting a blaze in a commercial apartment building, a large part of the brick wall fell on top of him, burying him under it. He had to be hospitalized due to inhalation of large amounts of smoke, silica, alumina, and other components of the brick wall. Subsequently, he worked in construction, laying down commercial water pipes for eight years.

General examination did not reveal any signs of connective tissue disease. His chest examination revealed bibasilar, fine, dry Velcro-like, mid to late inspiratory crackles. No other audible adventitious sounds were appreciated.

Investigation and treatment

Laboratory tests revealed a normal complete blood count (CBC); comprehensive metabolic panel (CMP), CRP, and ESR; negative autoantibody serology; negative fungal serology; normal angiotensin-converting enzyme (ACE) level; a negative hypersensitivity pneumonitis screen; and a negative TB-Gold QuantiFERON test. A spirometry test showed moderate restriction.

**Figure 1:**
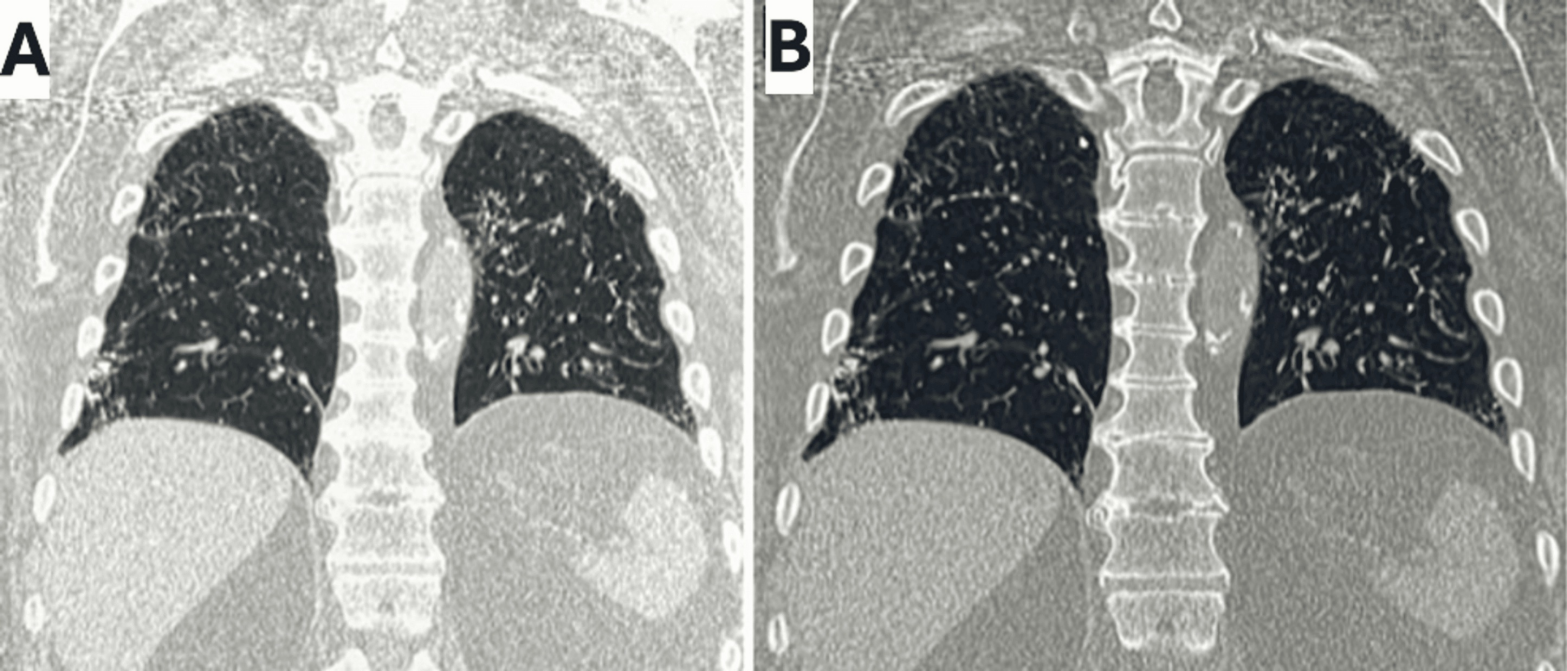
HRCT of the chest (coronal view) showing lung window (A) and bone window (B). Reveals extensive peripheral, bibasilar linear reticulations, interstitial thickening, and subpleural cystic changes consistent with advanced pulmonary fibrosis. Once again, branched calcified densities seen more prominently in the bone window (B). HRCT: high-resolution computed tomography

**Figure 2:**
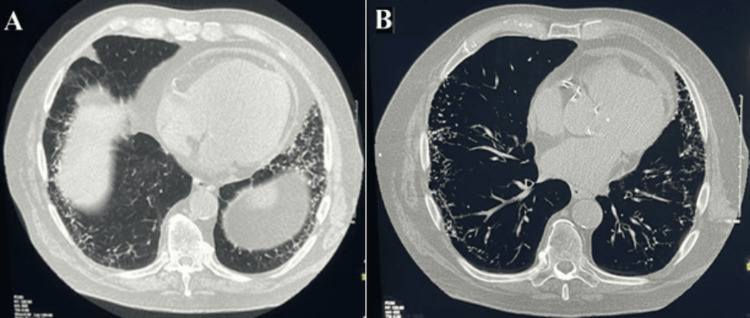
High-resolution computed tomography (HRCT) (axial view) normal window (A-B) of the chest displaying linear reticulations and interstitial thickening, with branched calcified densities seen in some areas of linear reticulation. Lung window settings (A) and bone window (B).

A high-resolution computed tomography (HRCT) scan (Figure 1) revealed extensive peripheral bibasilar linear reticulations, interstitial thickening, and subpleural cystic changes, consistent with advanced pulmonary fibrosis. Also noted were diffuse calcifications within areas of fibrosis. There were branched calcified densities in some areas of linear reticulations (Figures 1, 2). The pleura was normal without calcifications. No calcified granulomas or mediastinal nodal calcifications were present. No calcifications were noted in the liver or spleen. A bone scan did not reveal any uptake in the lung parenchyma.

Based on the clinical presentation as well as HRCT chest findings, a diagnosis of DPO associated with idiopathic pulmonary fibrosis was made. A lung biopsy was not performed due to the accompanying risks of surgery and the lack of usefulness of the biopsy findings associated with definitive alterations of treatment and prognosis pertaining to the underlying disease process.

The patient consented to the publication of his case on August 28, 2023, using a paper form. The patient was consented by Dr. Arun Adlakha.

## Discussion

Ossification is defined as the deposition of calcium in the collagen matrix seen with the presence of osteoblastic cells [1]. As previously noted, DPO is categorized as nodular and dendriform. The nodular type is associated with chronic heart failure and vascular disorders, especially in patients with a history of mitral stenosis [1]. Based on the literature, it is believed that the autolysis of extravasated red blood cells results in hemosiderin deposition, which triggers the process of fibrosis and hyalinization [1].

On the other hand, two possible hypotheses have been mentioned in the literature to describe the mechanism of dendriform pulmonary ossification. One hypothesis suggests that dendriform arises in a low pH and low oxygen environment brought on by recurrent pneumonia and lung fibrosis [4], where the lung fibroblasts and macrophages undergo metaplasia into osteoblasts and osteoclasts [1,4]. It is evident in patients with DPO, occurring in chronically injured lungs and lower lobes where ventilation and perfusion ratios are low, resulting in decreased oxygen perfusion and low pH [1]. The second hypothesis suggests that transforming growth factor-β produced by damaged epithelial cells stimulates ectopic bone formation in the lungs [4], mutations in transcription factors regulating bone morphogenic protein (BMPs), and Glast-expressing progenitor mesenchymal stem cells (MSCs). The latter have been shown to play an important role in the formation of chondrocytes and osteocytes [1]. Other mediators that have also been reported are interleukin-1 (IL-1) and interleukin-4 (IL-4) [5].

The dendriform is seen in association with a variety of underlying conditions such as chronic lung disease, pulmonary amyloidosis, histoplasmosis, sarcoidosis, and interstitial pulmonary fibrosis [5]. Intriguingly, an association between DM and DPO is also noted in the literature, stating that DM results in medial vascular calcification through the stimulation of osteogenesis resulting in mineralization due to oxidative stress, inflammation, and glycation end-products [1]. DPO is also associated with gastro-esophageal reflux disease (GERD) [1] due to chronic aspiration of gastric acid [6]. In our case, the patient had a past medical history of chronic diabetes and GERD, which can play a role in the pathogenesis of DPO, though further studies are needed to show a definitive correlation between these diseases and DPO. Our patient did present with bibasilar, dry Velcro-like inspiratory crackles, suggesting further investigation leading to the diagnosis of DPO in the setting of interstitial pulmonary fibrosis. Based on our patient’s HRCT findings, our patient presented with dendriform DPO.

Jungmann et al. [7] described a case of DPO in an 83-year-old man with a previous occupational history as a dentist, working at a dental prosthetic manufacturer. The patient was chronically exposed to silica due to his occupational history, which left him with symptoms of chest pain and partial pneumothorax [7]. The patient underwent video-assisted thoracoscopic surgery, and biopsies showed lamellar deposits of calcified osteoid material with marrow elements in the alveolar spaces and interstitium, concurring with DPO. Thus, it was concluded that occupational exposure to silica may have played a role in the pathogenesis of DPO [7]. Table 1 presents some of the other occupations, with associated occupational inhalational exposures that are likely determinants in the causation of DPO.

**Table 1:** Presents various risk factors, occupations with associated exposures playing a role in the causation of DPO. M: male; F: female; PY: pack years; COPD: chronic obstructive pulmonary disease; GERD: gastro-esophageal reflux disease; DPO: diffuse pulmonary ossification

Publication	Age (years)	Sex	Risk factors	Occupation
Polit et al. [1]	36	M	Childhood asthma, obese, non-smoker	Security guard in a hospital
Alami et al. [2]	75	M	COPD, former smoker (40 PY)	No professional or environmental allergen exposure
Sweidan et al. [4]	45	M	GERD	Veteran, served in the Gulf War for 7 months. Thereafter, worked for 8 months in a dusty warehouse in Iraq
Jungmann et al. [7]	83	M	Non-smoker	Dentist at a dental prosthesis manufacturer
Edahiro et al. [8]	35	M	Former smoker (6 PY)	Systems engineer
Matsuo et al. [9]	47	M	Former smoker (29 PY), spontaneous pneumothorax	Plumber for 17 years, then industrial waste disposal wearing a dust mask for 5 years
Carnevale et al. [10]	77	M	Former smoker (15 PY)	Former surveyor in a chemical company
Burkett et al. [11]	82	M	Former smoker (80 PY)	Retired truck driver
Harvey et al. [12]	47	M	None	Dental technician for 20 years with reported exposure to methylmethacrylate, quartz, cristobalite, chrome-cobalt, alginates, asbestos, and silica

DPO is most prevalent in men in their 40s-60s but has also been identified in younger men and women [5]. It has a predilection for lower lobes with posterior and lateral basal segments [5]. Patients usually present with non-specific signs and symptoms of dyspnea, non-productive cough, chest pain, and asthenia [7]. The most favorable imaging modality to diagnose DPO is HRCT. With the use of bone window settings (width, 2,500 HU; level, 500 HU) [1], multiple tiny calcifications even smaller than 1 mm can be visible and seen in lung bases especially in the posterior regions, with greater concentration in the subpleural parenchyma [7]. The HRCT can also detect the thickening of the lobular septa with the distribution of calcifications along the septa and around the centrilobular distal bronchioles [7]. In our case, HRCT helped to diagnose DPO, but a lung biopsy was not performed. Lung biopsy, when performed, will point to the histologic examination of the lung specimen, obtained during video-assisted thoracoscopy surgery (VATS), showing multiple foci of bone in a branching pattern, located within the alveolar airspaces, with some containing bone marrow elements [7-8]. As expected, our patient had a negative bone scan, with no uptake in the lung parenchyma or anywhere else within the thorax. The ectopic bone formation and deposition in the alveolar space and parenchyma with or without bone marrow elements are only evident on the histological examination of the biopsied pathological specimen.

DPO is considered an indolent disease with slow progression and deterioration over a decade [8]. However, a case study by Matsuo et al. [9] presented a patient with idiopathic DPO diagnosed at 30 years of age. The patient’s vital capacity (VC) was normal until 36 years of age but then severely deteriorated with progressively worsening symptoms of cough and dyspnea, which led the patient to be admitted. The patient’s chest X-ray revealed the progression of bilateral ground glass opacity, and pulmonary function tests showed the VC had decreased severely [9]. The patient was diagnosed with severe restrictive ventilatory impairment. With no medical treatment, the patient was evaluated for lung transplantation [9].

DPO is not due to the precipitation of phosphate and calcium in the lungs or hypercalcemia but is a heterotrophic bone formation within the pulmonary interstitium [5]. In our case, the differential diagnosis included, but was not limited to, pulmonary alveolar microlithiasis (PAM). PAM is a rare autosomal recessive, progressive pulmonary condition characterized by the accumulation of hydroxyapatite microliths within the lumen of alveolar spaces due to deficiency in the sodium-phosphate cotransporter NPT2B [1], secondary to mutations in the SLC34A2 gene [3]. PAM radiologically presents similarly to DPO with calcifications in the lower lobes but has familial association and is histologically characterized by concentrically laminated calcifications in the alveolar spaces and interstitium [1].

The treatment and prognosis of DPO are still unknown and remain difficult to determine, as there is no large published series that has shown any definitive treatment modality for this entity [2,4,8]. In most cases, symptoms remain stable with little to no progression for many years [4]. However, in certain cases, rapid progression of symptoms is noted that leads to debilitating consequences [9]. Current therapeutic measurements that target ossification are only experimental and aim to manage symptoms or complications [4]. Our patient has been prescribed the antifibrotic drug nintedanib (Ofev) to treat the underlying idiopathic pulmonary fibrosis.

## Conclusions

After a literature review, no cases were detected pertaining to the development of DPO in the setting of occupation as a firefighter. We hypothesize that chronic exposure to combustible trash mixtures, smoke, silica, alumina, and varieties of other particulate matter played a significant role in the development of heterotrophic bone formation in the parenchyma of the lungs. This case highlights the importance of occupational history and exposure to inhaled respiratory pollutants/insults in patients presenting with acute or chronic respiratory pathology of undetermined etiology.
